# Unravelling differences and hallmarks in suspected diffuse low-grade gliomas: a multicentre database study

**DOI:** 10.1093/braincomms/fcaf368

**Published:** 2025-09-27

**Authors:** Francesco Latini, Markus Fahlström, Alice Neimantaite, Tomás Gómez Vecchio, Alba Corell, Ole Solheim, Sasha Gulati, Peter Milos, Björn Sjögren, Lars Kjelsberg Pedersen, Anna Lipatnikova, Klas Holmgren, Rickard L Sjöberg, Henrietta Nittby Redebrandt, Gregor Tomasevic, Ruby Mahesparan, Øystein Vesterli Tveiten, Erik Thurin, Margret Jensdottir, Jiri Bartek, Maria Zetterling, Asgeir S Jakola

**Affiliations:** Department of Medical Sciences, Section of Neurosurgery Uppsala University Hospital, S-75185 Uppsala, Sweden; Department of Surgical Sciences, Molecular Imaging and Medical Physics, Uppsala University, S-75185 Uppsala, Sweden; Institute of Neuroscience and Physiology, Sahlgrenska Academy, University of Gothenburg, 40530 Gothenburg, Sweden; Institute of Neuroscience and Physiology, Sahlgrenska Academy, University of Gothenburg, 40530 Gothenburg, Sweden; Institute of Health and Care Sciences, Sahlgrenska Academy, University of Gothenburg, 40530 Gothenburg, Sweden; Institute of Neuroscience and Physiology, Sahlgrenska Academy, University of Gothenburg, 40530 Gothenburg, Sweden; Department of Neurosurgery, Sahlgrenska University Hospital, 40530 Gothenburg, Sweden; Department of Neuromedicine and Movement Science, Faculty of Medicine and Health Sciences, Norwegian University of Science and Technology, NTNU, 7491 Trondheim, Norway; Department of Neurosurgery, St. Olavs Hospital, Trondheim University Hospital, 7491 Trondheim, Norway; Department of Neuromedicine and Movement Science, Faculty of Medicine and Health Sciences, Norwegian University of Science and Technology, NTNU, 7491 Trondheim, Norway; Department of Neurosurgery, St. Olavs Hospital, Trondheim University Hospital, 7491 Trondheim, Norway; Department of Neurosurgery, Linköping University Hospital, 58185 Linköping, Sweden; Department of Biomedical and Clinical Sciences, Linköping University, 58185 Linköping, Sweden; Department of Neurosurgery, Linköping University Hospital, 58185 Linköping, Sweden; Department of Biomedical and Clinical Sciences, Linköping University, 58185 Linköping, Sweden; Department of Neurosurgery, University Hospital of North Norway, 9038 Tromsø, Norway; Institute of Neuroscience and Physiology, Sahlgrenska Academy, University of Gothenburg, 40530 Gothenburg, Sweden; Department of Clinical Sciences, Neuroscience, Umeå University, 90187 Umeå, Sweden; Department of Neurosurgery, University Hospital of Northern Sweden, 90187 Umeå, Sweden; Department of Clinical Sciences, Neuroscience, Umeå University, 90187 Umeå, Sweden; Department of Neurosurgery, University Hospital of Northern Sweden, 90187 Umeå, Sweden; Department of Neurosurgery, Skåne University Hospital, 22185 Lund, Sweden; Department of Neurosurgery, Skåne University Hospital, 22185 Lund, Sweden; Department of Clinical Medicine, Faculty of Medicine, University of Bergen, 5020 Bergen, Norway; Department of Neurosurgery, Haukeland University Hospital, Bergen 5020, Norway; Department of Clinical Medicine, Faculty of Medicine, University of Bergen, 5020 Bergen, Norway; Department of Neurosurgery, Haukeland University Hospital, Bergen 5020, Norway; Institute of Neuroscience and Physiology, Sahlgrenska Academy, University of Gothenburg, 40530 Gothenburg, Sweden; Department of Radiology, Sahlgrenska University Hospital, 40530 Gothenburg, Sweden; Department of Clinical Neuroscience, Karolinska Institutet, Stockholm, Sweden; Department of Neurosurgery, Karolinska University Hospital, 17176 Stockholm, Sweden; Department of Clinical Neuroscience, Karolinska Institutet, Stockholm, Sweden; Department of Neurosurgery, Karolinska University Hospital, 17176 Stockholm, Sweden; Department of Neurosurgery, Copenhagen University Hospital Rigshospitalet, 2100 Copenhagen, Denmark; Department of Medical Sciences, Section of Neurosurgery Uppsala University Hospital, S-75185 Uppsala, Sweden; Institute of Neuroscience and Physiology, Sahlgrenska Academy, University of Gothenburg, 40530 Gothenburg, Sweden; Department of Neurosurgery, Sahlgrenska University Hospital, 40530 Gothenburg, Sweden; Department of Neurosurgery, St. Olavs Hospital, Trondheim University Hospital, 7491 Trondheim, Norway

**Keywords:** diffuse low-grade gliomas, white matter, molecular profile, epilepsy, onco-functional trajectory

## Abstract

The natural history of suspected diffuse low-grade gliomas (DLGG) depends heavily upon the molecular status. To fully comprehend this integrated information preoperatively, a clinical phenotype incorporating both clinical and radiological information may be of value. We aimed to analyse this systematically in a large multicentre study to identify clinical/radiological phenotypes of DLGG molecular subgroups at the onset. Patients from nine Scandinavian centres, with confirmed World Health Organization (WHO) grade 2 at the time of diagnosis (according to the WHO 2016/2007 classification), known molecular status [isocitrate dehydrogenase (IDH) status and 1p19q codeletion status] and preoperative images of adequate quality, were analysed. MRI-based tumour volume segmentation was used to create a frequency map of their locations in the Montreal Neurological Institute space. A Brain-Grid (BG) system was also used for tumour invasiveness analysis. Variables were analysed for each subgroup of DLGG with regression analyses. A total of 235 patients were included in the study. The three molecular subgroups differed in age, tumour location, epileptic onset and cognitive status. Seizure onset was linked to the number of BG voxels and the A3C2S2 location in all three molecular subgroups. Cognitive deficits were related to increasing age (IDH-mutated oligodendrogliomas), female gender (IDH-wildtype) and tumour volume (oligodendrogliomas). Patients with IDH-mutated astrocytomas (*n* = 65) displayed younger age, left-sided fronto-insular preferential location, infiltration of anterior ventral inferior fronto-occipital fasciculus (IFOF) and external capsule, and seizure as the onset symptoms. Oligodendrogliomas (*n* = 116) were more often found in patients >40 years old, with frontal location, dorsal IFOF, frontal aslant tract and superior longitudinal fasciculus invasion, and seizures as the onset symptoms. IDH-wildtype astrocytomas (*n* = 54) displayed: age >40 years old, left-sided temporo-insular preferential location, invasion of posterior IFOF and cortico-spinal tract, cognitive deficits at onset and the infiltration of posterior left peri-insular voxel (A3C2S3) as a strong predictor of IDH-wildtype. Using an integrated clinico-radiological approach, we identified differences in age, clinical presentation, preferential location and white matter infiltration among specific molecular subgroups of suspected DLGG. The systematic combination of patient-specific variables (age/clinical onset) and tumour-specific features (sub-lobar preferential location) may be relevant to create future prediction models and to better understand the onco-functional trajectory already at the preoperative stage. Prediction models may benefit from combining information rather than, for instance, analysing images only.

## Teaser

In 235 patients with suspected diffuse low-grade glioma, clinico-radiological patterns varied by molecular subgroup: isocitrate dehydrogenase–mutated astrocytomas occurred in younger patients with left fronto-insular tumours; oligodendrogliomas in older patients with frontal tumours and seizures, and wildtype astrocytomas in older patients with temporo-insular tumours and cognitive deficits. Integrated features may improve preoperative prediction.

## Graphical Abstract

**Graphical Abstract:**
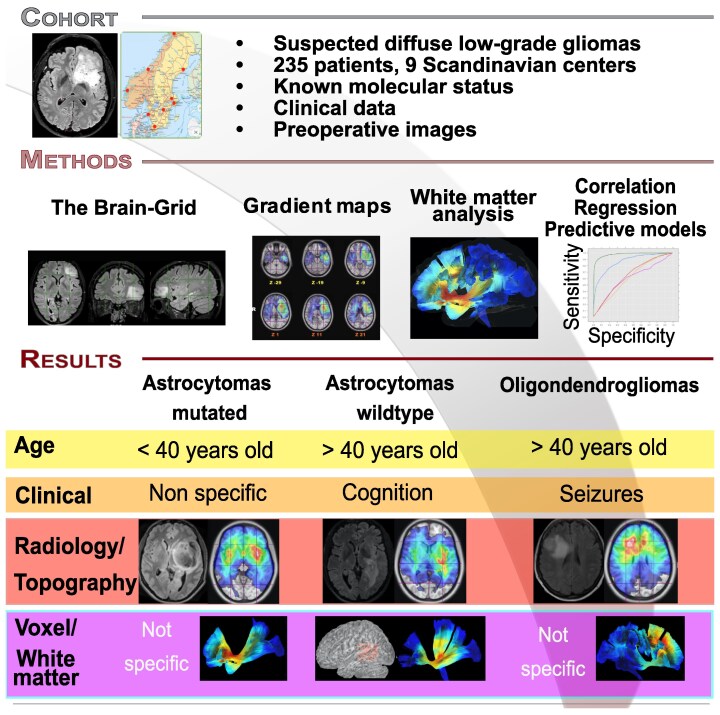

## Introduction

The radiological appearance and clinical course of grade 2 diffuse low-grade gliomas (DLGG) are diverse but rely heavily upon their molecular subtype.^1-5^

Suspected DLGG may display different preferential locations depending on their molecular subtype, various radiological features and different surgical implications.^6-8^ The initial tumour location, subcortical extension and clinical onset are the primary factors on which surgical management is dependent on.^1,4,9,10^

Variations can be observed regarding the radiological features of DLGG, with some tumours having distinct radiological borders while others showing more pronounced subcortical infiltration or even bilateral extension that can be considered negative prognostic factors.^2,11,12^

From the clinical perspective, DLGG patients are heterogeneous, with clinical presentation varying from true incidental findings to neurological and cognitive deficits.^1,11,13,14^

A limitation in predicting the clinical and radiological course is the historically insufficient focus on white matter infiltration. Tumour invasiveness through subcortical structures differs among DLGG subtypes and is difficult to quantify but largely impacts the surgical choice, the overall surgical results, the functional outcome and the overall survival (OS) of the patient.^9,15,16^

Differences among DLGG molecular subgroups in terms of preferential location, invasiveness and OS have been described.^13,17-20^ However, an integrated approach considering symptom onset, radiological features and molecular status of the tumours has not been reported. The differences in clinico-radiological phenotypes of suspected DLGG might be of value in clinical management.

In this study, we investigate patient- and tumour-specific variables available preoperatively in a multicentre Scandinavian cohort. Our aim was to define clinico-radiological phenotypes specific to molecular subgroups of DLGGs.

## Materials and methods

### Study design and included patients

The study is part of a collaborative, multicentre project including nine neurosurgical departments performing glioma surgery in Norway and Sweden.^10^ Patients screened for inclusion were adults aged 18 years or above who underwent primary surgery (biopsy or resection) of a histopathologically verified supratentorial diffuse WHO grade 2 glioma in the time period 2012–2017.^21,22^

**Figure 1:**
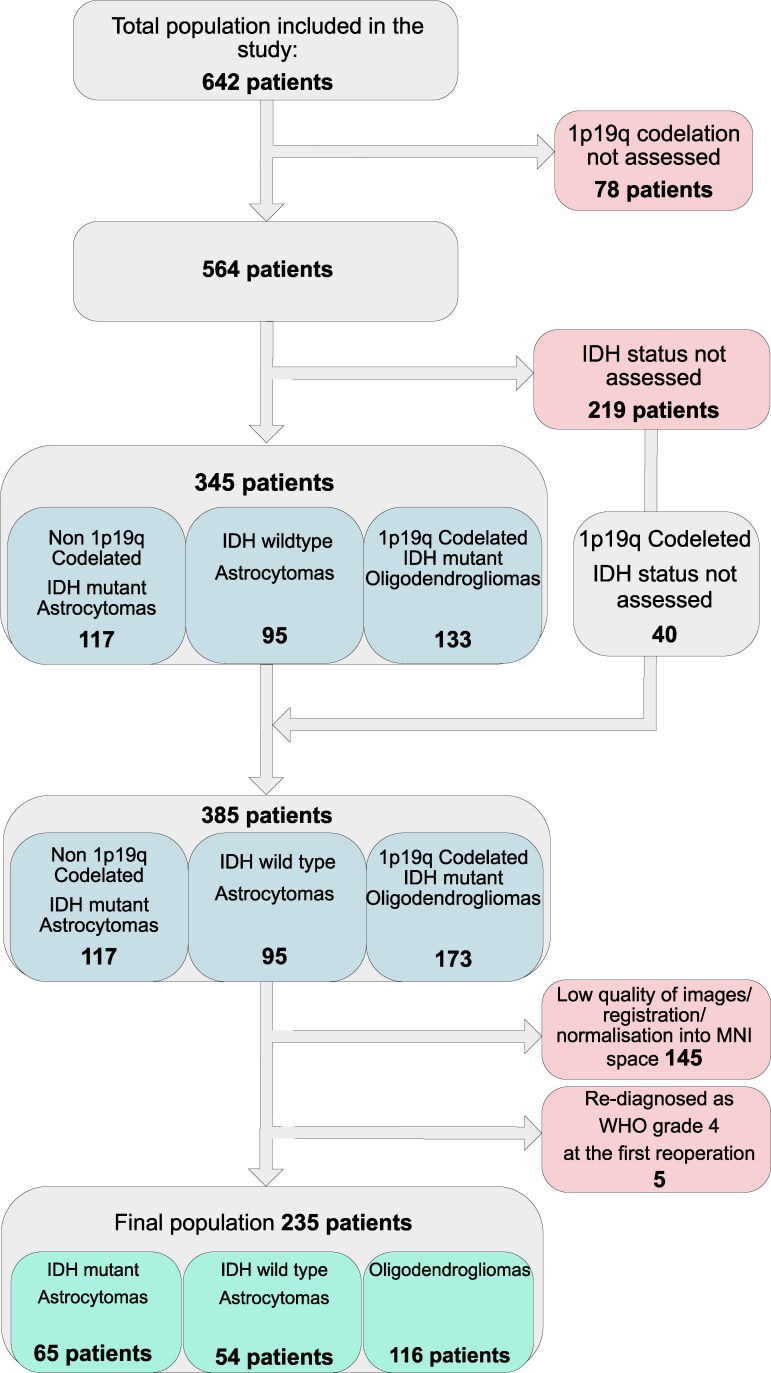
**Flow chart of the study population.** The figure displays the flow chart of the study population from the original cohort of 642 patients. The actual study population is based on the histopathological diagnoses at primary surgery and molecular genetic status within histopathological subtypes based on available mutational assessments of IDH and 1p/19q. Patients with confirmed 1p19q codeletion were included in the study cohort based on the absence of IDH mutation in the literature. The patients were, therefore, included as oligodendrogliomas. A total population of 235 was included in the present study. *IDH, isocitrate dehydrogenase; MNI, Montreal Neurological Institute; WHO, World Health Organization*.

From the initial population (642 patients), only those with confirmed molecular diagnosis, including isocitrate dehydrogenase (IDH) status (mutated, IDH-m; or wildtype, IDH-wt), and loss of heterozygosity (LOH) for chromosome arms 1p and 19q (LOH 1p19q), were further included for analysis. The flow diagram specifying the actual population is displayed in Fig. 1.

### Patients data

Pseudonymized clinical and radiological data were retrieved from the electronic health records (EHR) at each institution or collected from research projects conducted locally. Clinical data included patient demographics, the Karnofsky performance score (KPS), symptoms at presentation, histopathological tumour grade, IDH status and 1p19q codeletion status, main tumour location, tumour largest diameter and presumed eloquence based on the University of California San Francisco (UCSF) criteria.^23^ Outcome data included death status at the last follow-up of the study in 2021.

### Radiological assessment and DLGG segmentation

Morphological MRI findings with high signal intensity on T2-weighted or T2-weighted fluid attenuated inversion recovery (FLAIR) were re-evaluated by the first author of this study to collect information regarding the radiological borders (sharp or diffuse) and then re-analysed using the Brain-Grid (BG) system.^24^ The tumour volume was segmented on T2 FLAIR or T2 TSE images in 3D Slicer with the technique previously described.^8,25^

Furthermore, T2 TSE or T2 FLAIR images were normalized to MNI space. Tumour segmentations and T2 or FLAIR images were transformed to the MNI space by applying the transformation matrix generated during T1 or T1c registration to the MNI space (T1 symmetric MNI 09a). All transformations to the MNI space were individually controlled for errors or unexpected deformations by two different raters with experience in glioma image analysis. Image registration was performed using 12 parameter affine transformations in FSL’s Functional Magnetic Resonance Imaging of the Brain Linear Image Registration Tool (FLIRT).^26^ For the analysis of tumour borders, we considered either bulky/regular lesions, with well-defined/sharp margins (without any finger-like hyperintense signals on T2 TSE or T2 FLAIR sequences), or tumour margins with unclear and irregular signal intensity on T2 FLAIR sequences, which were considered diffuse, as previously described.^14,17^

### Assessment of tumour preferential location

Segmented tumours in MNI standard space were combined into three different tumour occurrence weighted maps depending on the molecular status of the tumour: astrocytomas IDH-m, astrocytomas IDH-wt or oligodendroglioma, and normalized to the total number of tumours within each group, respectively. Using the tumour occurrence weighted maps, we qualitatively compared the preferential locations of tumours with different molecular status.^17,27^

Based on previous results, we selected six major white matter bundles, which showed regional differences of infiltration frequency.^16,17^ The arcuate fasciculus (AF), the anterior indirect component of superior longitudinal fasciculus (SLF2-3), inferior fronto-occipital fasciculus (IFOF), the cortico-spinal tract (CST), frontal aslant tract (FAT) and the cingulum (Ci) were reconstructed within the Human Connectome Project (HCP-1065) template following the anatomical criteria already published with the Brain Grid tractographic reference atlas^17,24^ and overlaid with tumour occurrence weighted map for each subtype.^16^

### Sub-lobar analysis of tumour location and extension

Sub-lobar analysis of tumour location and subcortical extension was performed using the BG system.^14,17,24^ Briefly, the BG was constructed by three axial lines, two coronal lines and three sagittal lines, whereas the intersection of these lines creates 48 grid voxels as sub-lobar classification system, including also white matter connectivity information through a tractographic reference atlas.^24^ For each segmented DLGG, the number of grid voxels, i.e. DLGG extension and location, was recorded. The quantitative and qualitative analysis of the tumour presence within each BG voxel was performed in MNI space using DSI Studio (DSI Studio, https://dsi-studio.labsolver.org/).

### Statistical analysis

The statistical analysis was performed in several steps as outlined below. The Kolmogorov–Smirnov test was performed on all variables included for statistical analysis to test for normality. Derived *P*-values are two-sided and presented as exact values or *P* < 0.001, with *P* < 0.05 being considered significant. SPSS version 29.0 (SPSS, Inc., Chicago, IL) was used for statistical analysis.

Frequency distributions and summary statistics were calculated for the whole patient population and for each molecular status, respectively. To test whether any differences were present between DLGG subgroups for a given patient and DLGG-related variable, a Kruskal–Wallis test for independent samples on non-parametric continuous variables and Pearson’s chi-square test or Fisher’s exact test were used for categorical variables. Moreover, a median test (non-parametric *k* samples) was performed for the variables tumour volume and number of BG voxels. False discovery rate (FDR) with Benjamini–Hochberg correction method was applied to the results.

### *Post hoc* analysis

A binary logistic regression model was performed on clinical variables, which significantly differed between the molecular status groups as dependent variables. Demographic and radiological variables and the BG voxels in which the DLGG was most present were included as independent variables. FDR correction was not applied in this case, but all the variables were entered into a multivariable analysis to confirm independent predictors of the outcome measures.^28^

### Predictors of the final diagnosis

To identify possible predictors of histopathological diagnosis (IDH-m astrocytomas, oligodendrogliomas or IDH-wt astrocytomas), we analysed the distinctive variables among the groups (age, seizure onset, cognitive impairment, laterality and number of BG voxels, and the invasion of the most frequently infiltrated BG voxels) and performed a multinomial regression analysis. A test for collinearity was performed before the regression. The diagnoses of the three groups were included as dependent variables, and the prognostic variables were encoded as dichotomous or categorical variables as previously described.^14^ Receiver operating characteristic (ROC) curves were then obtained for the significant variables in each molecular subgroup to obtain an estimation of the prediction model (area under the curve, AUC). Positive predictive value (PPV) and negative predictive value (NPV) were calculated for the cumulative prognostic model according to previous publications^29^ using the following formulas:


$$\begin{array}{rl} & \mathrm{PPV}=\mathrm{Sensitivity}\;\mathrm{timesPrevalence}\\ & \mathrm{Sensitivity}\;\mathrm{timesPrevalence}+(1-\mathrm{Specificity})\times (1-\mathrm{Prevalence}) \end{array}$$

$$\begin{array}{rl} & \mathrm{NPV}=\mathrm{Specificity}\;\mathrm{times}(1-\mathrm{Prevalence})\\ & \mathrm{Specificity}\;\mathrm{times}(1-\mathrm{Prevalence})+(1-\mathrm{Sensitivity})\times \mathrm{Prevalence} \end{array}$$

### Ethics and approvals

The study was conducted according to the Declaration of Helsinki, and it was approved by the regional committee of Western Sweden (EPN reference 705/17) and the Regional Committee for Medical and Health Research Ethics in Central Norway (REC reference 2017/1780). The need for informed consent was waived by the committees.

## Results

### Patient characteristics

A summary of the patient population is displayed in Tables 1 and 2. Fifty per cent of the cases were oligodendrogliomas, while 23% of the patients received a diagnosis of IDH-wt astrocytomas. In 62% of the patients, there was a diffuse radiological border, while 23% had minor (patchy/faint) contrast enhancement. The most common location was frontal (59.9%). Concerning clinical presentation, seizures (57.4%) were the most common symptom, followed by headache (19.1%), while 15.7% were asymptomatic.

**Table 1:** Summary of demographic and radiological/topographical data

Patient population characteristics	
Total number of patients, *n*	235
Demographic	
Age, years, mean ± SD	42 ± 15
Male/female, *n* (%)	125 (53)/110 (47)
Radiological	
Radiological border, S/D, *n* (%)	89 (38)/146 (62)
Volume, ml, mean ± SD	55 ± 32
Contrast, Y/N, (%)	23.4/76.6
BG voxels, median (IQR)	8 (4–11)
Laterality, L/R/B, %	48.9/46.0/5.1
Main tumour location	
Frontal, %	57.9
Temporal, %	19.1
Parietal, %	8.5
Occipital, %	2.6
Insula, %	7.2
Central/basal ganglia, %	4.7
Multifocal, Y *n*/%	14/6

B, bilateral; D, diffuse; ICP, intracranial pressure; IQR, interquartile range; L, left; *n*, number of; *N*, No; *n*, number; R, right; S, sharp borders; SD, standard deviation; Y, yes, RDT, radiotherapy.

**Table 2:** Summary of clinical, surgical and outcome data

Patient population characteristics	
Clinical	
Seizure, Y *n*/%	135/57.4
Cognitive deficit, Y *n*/%	23/9.8
Motor deficit, Y *n*/%	28/11.9
Language deficit, Y *n*/%	18/7.7
Visual deficit, Y *n*/%	16/6.8
Headache/ICP-related problems, Y *n*/%	45/19.1
Asymptomatic, Y *n*/%	37/15.7
Clinical deterioration before surgery, Y *n*/%	9/3.8
Karnofsky performance scale >90, Y *n*/%	175/74.5
Neuropsychological assessment, Y/*N* %	22.6/77.4
Neuropsychological impairment if registered, Y *n*/%	20/8.5
Surgical	
Eloquence, Y *n*/%	146/62.1
Biopsy only, %	18.3
Resection, %	81.7
Oncological treatment	
Early radiotherapy *n*/%	87/37
Early chemotherapy *n*/%	58/24.7
Late radiotherapy *n*/%	45/19.1
Late chemotherapy *n*/%	66/28.1
Outcome	
Dead, Y/*N*, %	16.6/83.4
Days from surgery to death, mean ± SD	802 ± 661

B, bilateral; D, diffuse; ICP, intracranial pressure; IQR, interquartile range; L, left; *n*, number of; *N*, No; *n*, number; R, right; S, sharp borders; SD, standard deviation; Y, Yes; RDT, radiotherapy.

### Between-group comparisons

As seen in Tables 3 and 4, a significant difference in the age of the groups was displayed. Patients with astrocytomas IDH-m were younger (38 ± 13) than those with oligodendrogliomas (42 ± 14, *P* < 0.05) and astrocytomas IDH-wt (48 ± 18, *P* < 0.01). Oligodendrogliomas were younger than astrocytomas IDH-wt (48 ± 18, *P* < 0.05). No differences in terms of preoperative volume, radiological borders or presence of contrast enhancement were seen between the groups (Tables 3 and 4).

**Table 3:** Between-group analysis for demographic and radiological, variables

Variables	Astrocytomas IDH-m	Astrocytomas IDH-wt	Oligodendrogliomas	Test	*P*-value	FDR
Number of patients, *n*(%)	65 (27.7)	54 (23)	116 (49.4)			
Demographics						
Age, years, mean ± SD	38 ± 13	48 ± 18	42 ± 14	KW	0.006*	0.022*
Male/female, *n* (%)	36 (55.4)/29 (44.6)	28 (51.9)/26(48.1)	61 (52.6)/55(47.4)	Chi square	0.913	0.925
Radiological						
Radiological border, S/D, *n*(%)	26(40)/39(60)	14 (25.9)/40 (74.1)	48 (41.4)/68(58.6)	Chi square	0.135	0.212
Volume, ml, mean ± SD	60 ± 64	53 ± 61	47 ± 43	KW	0.682	0.750
Contrast, Y/*N*, *n*%	9(13.8)/56(86.2)	17(29.8)/40(70.2)	29 (25)/87(75)	Chi square	0.076	0.139
BG voxels, median (IQR)	7/3–13	8.5/4–12	8/4–10	Median t	0.035	0.085
Laterality, L/R/B, %	35/28/2	24/23/7	56/57/3	Chi square	0.049	0.107
Main tumour location				Chi square	<0.001*	0.022*
Frontal, *n* (%)	35 (53.8)	20 (37.0)	81 (69.8)			
Temporal, *n* (%)	16 (24.6)	16 (29.6)	13 (11.2)			
Parietal, *n* (%)	9 (13.8)	3 (5.6)	8 (6.9)			
Occipital, *n* (%)	0	1 (1.9)	5 (4.3)			
Insula, *n* (%)	5 (7.7)	4 (7.4)	8 (6.9)			
Central/basal ganglia, *n* (%)	0	10 (18.5)	1 (0.9)			
Multifocal, Y, *n* (%)	2 (3.1)	5 (9.2)	7 (6)	Chi square	0.228	0.316

Significant differences between groups are marked with asterisks if *P*-values resulted <0.05 after correction for multiple comparisons.

B, bilateral; D, diffuse; IQR, interquartile range; KW, Kruskal–Wallis; L, left; *n*, number of; *N*, No; R, right; S, sharp borders; SD, standard deviation; Y, yes; FDR: false discovery rate correction.

**Table 4:** Between-group analysis for clinical, surgical and outcome variables

Variables	Astrocytomas IDH-m	Astrocytomas IDH-wt	Oligodendrogliomas	Test	*P*-value	FDR
Number of patients, *n*(%)	65 (27.7)	54 (23)	116 (49.4)			
Clinical						
Seizure, Y/*N*, *n* (%)	34(52.3)/31(47.7)	24(44.4)/30(55.6)	77 (66.4)/39(33.6)	Chi square	0.016*	0.044*
Cognitive deficit, Y/*N*, *n* (%)	6 (9.2)/59 (90.8)	14(25.9)/40(74.1)	3(2.6)/113(97.4)	Chi square	<0.001*	0.022*
Motor deficit, Y/*N*, *n* (%)	7(10.8)/58 (89.2)	10 (18.5)/44(81.5)	11(9.5)/105 (90.5)	Chi square	0.226	0.316
Language deficit, Y/*N*, *n* (%)	5 (7.7)/60(92.3)	8(14.8)/46(85.2)	5(4.3)/111(95.7)	Chi square	0.056	0.112
Visual deficit, Y/*N*, *n* (%)	4(6.2)/61(93.8)	7 (13)/47(87)	5(4.3)/111(95.7)	Chi square	0.110	0.186
Headache/ICP problems, Y/*N*, *n*(%)	17(26.2)/48(73.8)	10(18.5)/44(81.5)	18(15.5)/98(84.5)	Chi square	0.216	0.316
Asymptomatic, Y/*N*, *n* (%)	9 (13.8)/56(86.2)	9(16.7)/45(83.3)	19(16.4)/97(83.6)	Chi square	0.884	0.925
Karnofsky performance scale >90, Y/*N*, *n* (%)	46(70.8)/16(29.2)	36 (66.7)/18(33.3)	90 (77.6)/26 (24.1)	Chi square	0.309	0.378
Neuropsychological impairment^a^, Y/*N*, *n* (%)	7 (10.8)/14(21.5)	2(5.6)/3(5.6)	11(9.5)/17(14.7)	Chi square	0.903	0.925
Surgical						
Eloquence, Y/*N*, *n* (%)	37 (57.8)/27(42.2)	36 (66.7)/18(33.3)	74 (63.7)/42(36.3)	Chi square	0.593	0.686
Biopsy only, *n* (%)	6(9.2)	26 (48.1)	9(9.5)	Chi square	<0.001*	0.022*
Resection, *n* (%)	59(90.8)	28(51.9)	105(90.5)	Chi square	<0.001*	0.022*
Oncological treatment (SOC)						
Early radiotherapy *n*/%	24/36.9	26/48.1	37/31.9			
Early chemotherapy n/%	16/24.6	7/13	35/30.2			
Late radiotherapy *n*/%	18/27.7	5/9.3	22/19			
Late chemotherapy *n*/%	20/30.8	19/35.2	27/23.3			
Outcome						
Dead, Y/*N*, *n* (%)	6 (9.2)/59 (90.8)	23(42.6)/31(57.4)	10(8.6)/106(91.4)	Chi square	<0.001*	0.022*

Significant differences between groups are marked with asterisks if *P*-values resulted <0.05 after correction for multiple comparisons.

^a^When registered; ICP, intracranial pressure; IQR, interquartile range; KW, Kruskal–Wallis; L, left; *n*, number of; *N*, No; R, right; S, sharp borders; SD, standard deviation; Y, Yes; FDR, false discovery rate correction; SOC, standard of care.

### Preferential location

**Figure 2:**
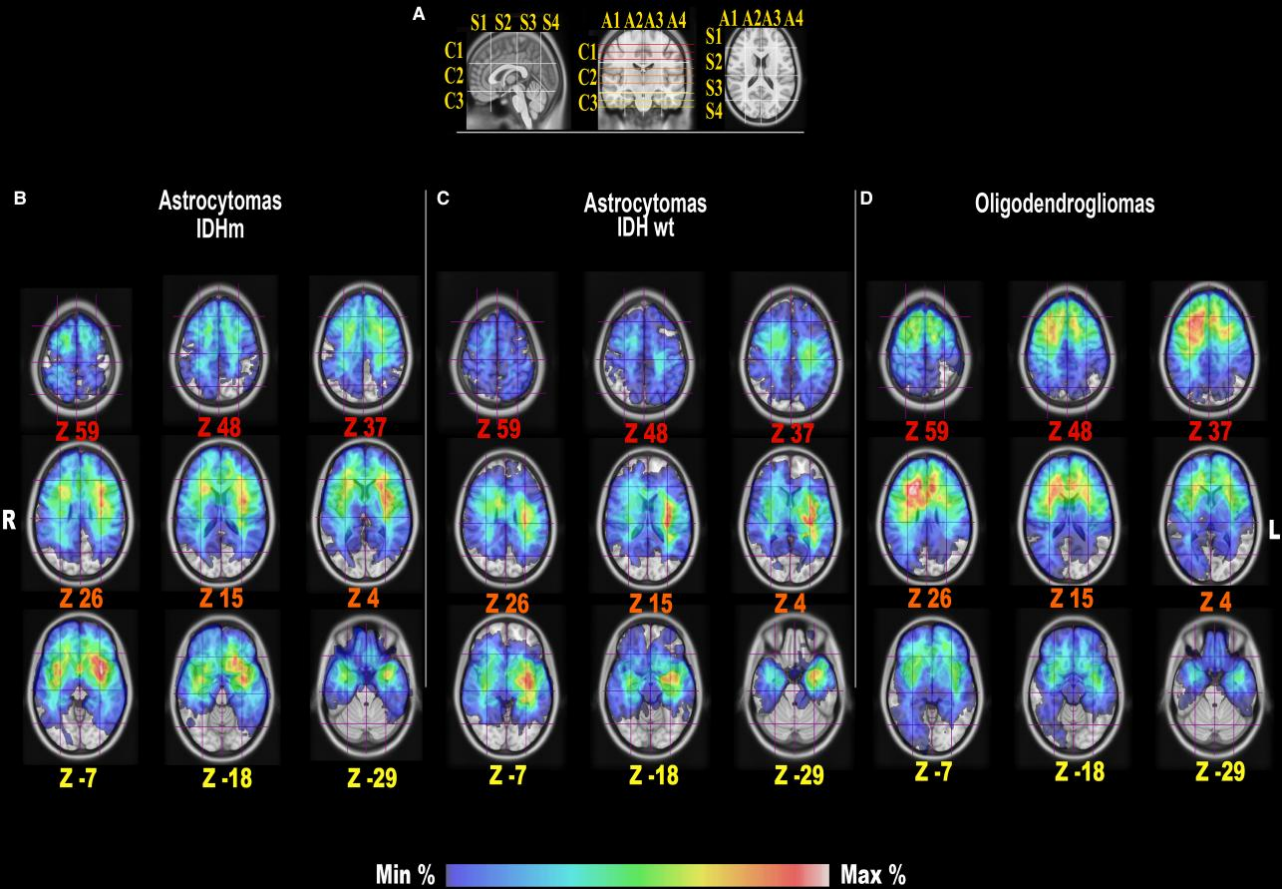
**Frequency of tumour infiltration.** The image shows the gradient maps reconstructed from the fusion of each tumour region within the MNI space (*Z* coordinates for each slice). In **A**, the BG system used as a reference for BG voxel count with sagittal, coronal and axial projections of the BG lines. In the lower part, the frequency of tumour location for the IDH-m astrocytomas (**B**), IDH-wt astrocytomas (**C**) and oligodendrogliomas (**D**) is colour graded (blue min, to red-white, as maximum in the gradient scale) according to the rate of voxel infiltration. The results provided are based on Min: minimum of infiltration (1.5%) and Max: maximum of infiltration (56.9%) frequency of each microvoxel. Regarding the BG voxel infiltration per each group, the raw data is displayed into Supplementary Table 1. The sample size was 65 for astrocytomas IDH-m, 54 for astrocytomas IDH-wt and 116 for oligodendrogliomas. *IDH, isocitrate dehydrogenase; m, mutation; wt, wild type; R, right; L, left, Min, minimum infiltration frequency; Max, maximum infiltration frequency.*

Preferential location differed generally between mutational subgroups. A difference in the predominant location among the different subgroups was identified (Fig. 2). IDH-m astrocytomas displayed the hot spot for infiltration of the anterior insular region, with the BG voxel A3C2S2 being infiltrated in 49.2% of the cases and A2C2S2 infiltrated in 49% of the cases. Astrocytomas IDH-wt showed a hot spot in the left posterior temporo-insular region, with A3C2S2 being infiltrated in 55.6% and A3C2S3 in 53.7% of the cases. Oligodendrogliomas infiltrated with the highest frequency into the bifrontal subcortical regions, with a significant right-sided infiltration with A3C2S2 in 56.9% and A2C2S2 in 51.7%. A summary of the different infiltration frequencies is displayed in Supplementary Table 1. The two most often infiltrated voxels for each subgroup were used for further topographical analyses.

**Figure 3:**
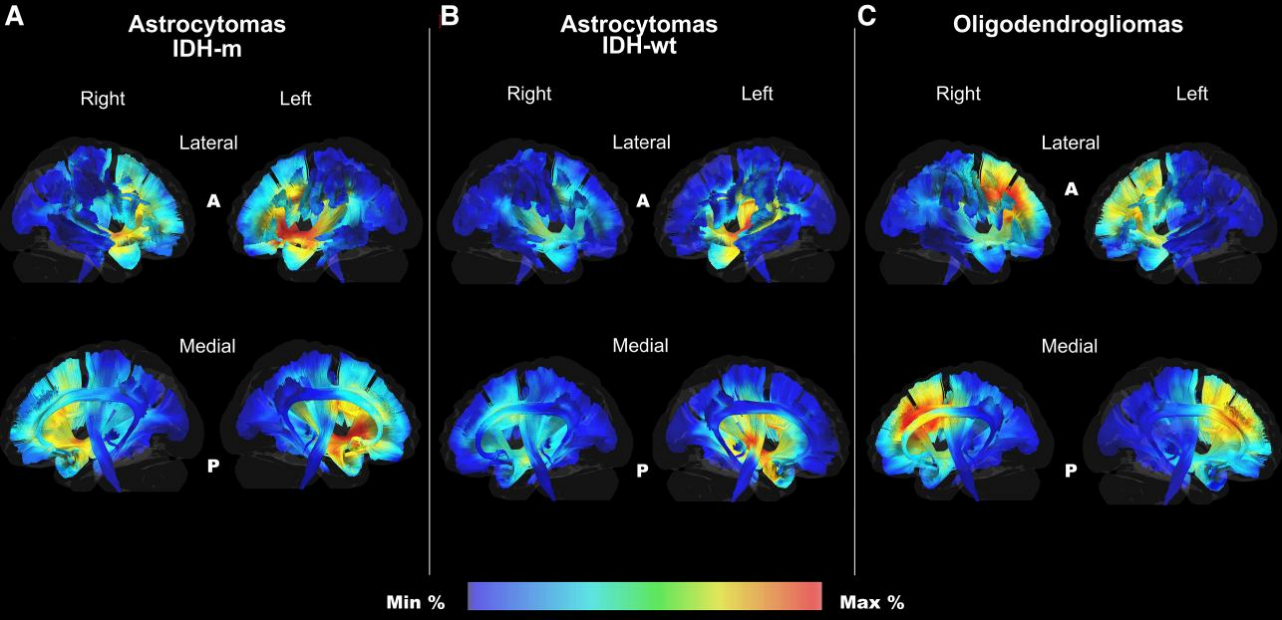
**Frequency of white matter infiltration.** The figure shows the probabilistic infiltration map obtained merging the tumour volumes of each molecular subgroup within MNI space. The AF, SLF2-3, IFOF, CST, FAT and Ci were reconstructed within the MNI space and merged with the gradient map for IDH-m astrocytomas in **A**, IDH-wt astrocytomas in **B** and oligodendrogliomas in **C**. The right and left sides of the reconstructed white matter bundles are displayed with a colour gradient, with red spectrum indicating the higher frequency of infiltration per voxel for each subgroup. The results provided are based on Min: minimum of infiltration (1.5%) and Max: maximum of infiltration (56.9%) frequency of each microvoxel. Regarding the BG voxel infiltration per each group, the raw data are displayed into Supplementary Table 1. The sample size was 65 for astrocytomas IDH-m, 54 for astrocytomas IDH-wt and 116 for oligodendrogliomas. *IDH, isocitrate dehydrogenase; m, mutation; wt, wild type; R, right; L, left; A, anterior; P posterior*.

In terms of white matter analysis, the left IFOF and UF displayed the highest infiltration probability for IDH-m astrocytomas, while the IDH-wt astrocytomas displayed a higher infiltration of the central regions of the IFOF and CST. Oligodendrogliomas showed a higher infiltration of the IFOF, AF and FAT, especially on the right side (Fig. 3).

### Clinical presentation

The clinical presentation differed among the three subgroups. Oligodendrogliomas displayed seizure as the first sign with respect to both astrocytomas IDH-m and astrocytomas IDH-wt (*P* < 0.05). Cognitive deficit was the distinctive sign for astrocytomas IDH-wt compared with both oligodendrogliomas and astrocytomas IDH-m (*P* < 0.05, Table 4).

The number of BG voxels and the infiltration of the A3C2S2 were common factors linked to seizures in all three groups (*P* < 0.05). Eloquent location according to Chang was a predictor of epileptic onset in IDH-m astrocytomas and oligodendrogliomas (*P* < 0.05). Radiological borders at FLAIR, gender differences and specific sub-lobar locations were associated with a risk of seizure onset among the three groups.

The presence of cognitive deficits was related to increasing age in both IDH-m astrocytomas and oligodendrogliomas (*P* < 0.05), while the female gender was a specific finding in the IDH-wt group (*P* < 0.05). Tumour volume was a specific predictor for cognitive deficits in oligodendrogliomas. The female gender was the only independent factor predicting cognitive deficits at the onset in the IDH-wt group.

The complete regression analysis related to clinically different presentation among subgroups is displayed in Tables 5 and 6.

**Table 5:** Binomial regression analysis for seizures as clinical onset symptom

Analysed variables	Astrocytomas IDH-m			Astrocytomas IDH-wt			Oligodendrogliomas		
	*P*	*OR*	*CI*	*P*	*OR*	*CI*	*P*	*OR*	*CI*
*Univariable analysis* * *Seizure*									
Gender M	0.198	0.611	0.289–1.294	0.395	0.625	0.212–1.846	0.006	2.235	1.262–3.960
Age	0.377	1.005	0.993–1.018	0.130	1.024	0.993–1.056	0.005	1.012	1.004–1.021
Radiological border D	0.631	1.167	0.622–2.190	0.008*	17.588	1.094–147.761	0.333	1.267	0.785–2.044
Volume	0.083	1.005	0.999–1.012	0.016*	1.016	1.003–1.029	0.076	1.005	0.999–1.010
Brain-Grid voxels	0.047*	1.053	1.01–1.109	0.023*	1.150	1.019–1.297	0.008*	1.061	1.015–1.109
Eloquent Y	0.037*	2.083	1.047–4.147	0.087	2.906	0.857–9.857	<0.001*	2.650	1.584–4.432
A2C1S2	0.410	1.675	0.491–5.714	0.709	0.783	0.217–2.826	0.852	1.090	0.441–2.692
A2C2S1	0.848	1.121	0.350–3.585	0.390	1.852	0.455–7.534	0.850	1.094	0.431–2.776
A2C2S2	0.162	1.667	0.815–3.409	0.091	2.615	0.858–7.970	0.006*	2.158	1.253–3.718
A2C2S3	0.232	1.833	0.678–4.957	0.529	1.429	0.470–4.340	0.028*	2.500	1.101–5.676
A3C2S2	0.039*	2.200	1.042–4.646	0.047*	3.176	1.017–9.915	0.015*	1.870	1.127–3.102
A3C2S3	0.258	2.000	0.602–6.642	0.275	1.831	0.618–5.425	0.493	1.375	0.553–3.418
*Multivariable analysis* * *Seizure*									
Gender M	0.014*	0.162	0.038–0.694						
Eloquent Y	0.017*	6.653	1.405–31.499						
Radiological borders D				0.016*	46.631	2.023–1074.65	0.003*	0.227	0.084–0.613
A3C2S2	0.030*	8.295	1.234–55.774						
A3C2S3				0.048*	0.045	0.002–0.970			

Seizures were used as dependent variables based on the different incidence among the three groups. Demographic, radiological and topographical variables in the three subgroups were included as test variables.

Multivariable regression analysis with proportional hazards modelling (forward conditional) was performed to assess the relative and independent prognostic capacity of each parameter and to avoid overestimation of significant results at the univariate analysis (*statistically significant for *P*  *<* 0.05).

M, male; Y, yes; OS, overall survival; HR, hazard risk; CI, confidence interval; D, diffuse.

**Table 6:** Binomial regression analysis for the cognitive deficits as clinical onset symptoms

Analysed variables	Astrocytomas IDH-m			Astrocytomas IDH-wt			Oligodendrogliomas		
	*P*	*OR*	*CI*	*P*	*OR*	*CI*	*P*	*OR*	*CI*
Univariable analysis * Cognitive deficits									
Gender M	0.781	1.269	0.236–6.816	0.027*	0.202 (−)	0.049–0.836	0.510	2.264	0.200–25.685
Age	0.013*	1.084	1.017–1.155	0.059	1.037	0.999–1.077	0.027*	1.205	1.022–1.420
Radiological border D	0.727	1.371	0.232–8.092	0.656	1.391	0.325–5.947	0.775	1.424	0.125–16.169
Volume	0.258	1.007	0.995–1.018	0.297	1.005	0.996–1.015	0.009*	1.019	1.005–1.033
Brain-Grid voxels	0.096	1.118	0.980–1.276	0.129	1.088	0.976–1.213	0.067	1.175	0.989–1.395
Eloquent Y	0.918	1.103	0.171–7.101	0.661	1.346	0.356–5.086	0.998	0.043	0.013–0.136
A2C1S2	0.479	0.485	0.065–3.603	0.083	0.199	0.032–1.238	0.800	0.698	0.043–11.277
A2C2S1	0.126	4.687	0.649–33.841	0.552	1.690	0.300–9.506	0.951	1.091	0.067–17.673
A2C2S2	0.968	1.034	0.193–5.549	0.764	1.206	0.354–4.115	0.605	1.897	0.167–21.513
A2C2S3	0.676	1.467	0.243–8.835	0.448	0.600	0.160–2.248	0.708	1.593	0.139–18.255
A3C2S2	0.379	2.214	0.376–13.034	0.172	2.500	0.671–9.310	0.997	0.048	0.015–0.152
A3C2S3	0.905	0.873	0.092–8.237	0.070	3.382	0.905–12.638	0.053	11.294	0.970–131.567
*Multivariable analysis* * *cognitive deficits*									
Gender M				0.018a*	0.116	0.019–0.694			

Cognitive deficits were used as dependent variables based on the different incidence among the three groups. Demographic, radiological and topographical variables in the three subgroups were included as test variables.

Multivariable regression analysis with proportional hazards modelling (forward conditional) was performed to assess the relative and independent prognostic capacity of each parameter and to avoid overestimation of significant results at the univariate analysis (*statistically significant for *P*  *<* 0.05).

M, male; Y, yes; OS, overall survival; HR, hazard risk; CI, confidence interval; D, diffuse.

### Clinical management

Astrocytomas IDH-wt underwent biopsy significantly more frequently than resection as a first strategy than the other two groups. Mortality was higher in astrocytomas IDH-wt group than the other two groups (*P* < 0.001). The distribution of demographics, clinical, radiological, tumour topography and outcome measures for each subgroup of DLGG is displayed in Tables 3 and 4.

### Predictors of the final diagnosis

**Figure 4:**
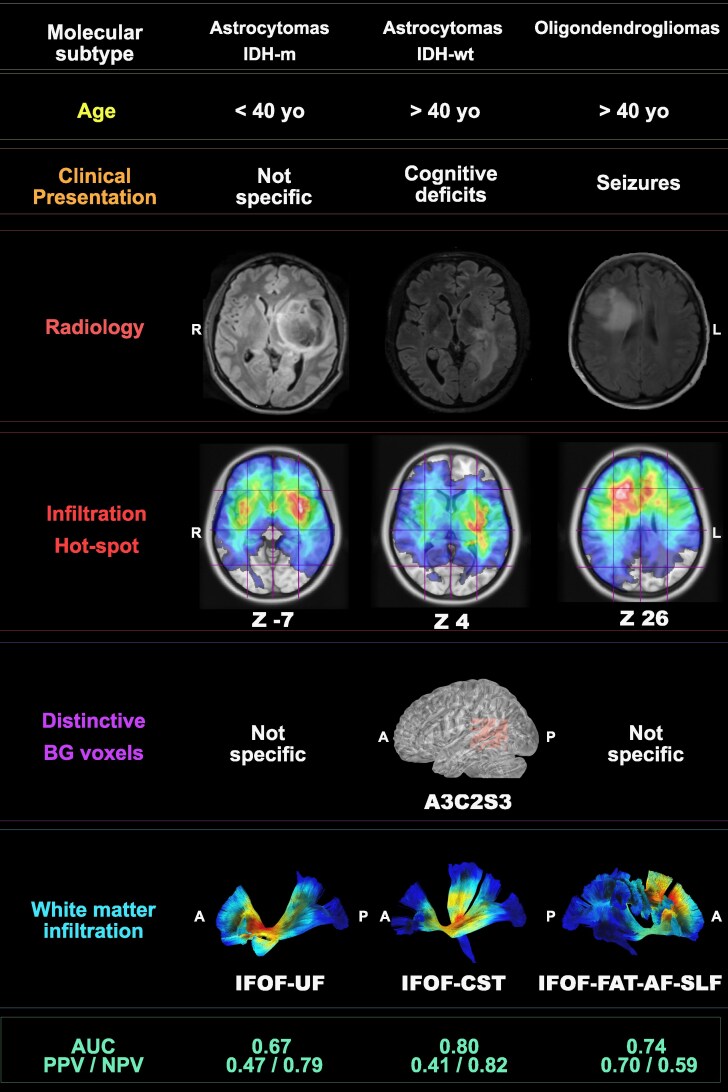
**Tumour hallmarks.** The illustration shows a summary of the differences and distinctive hallmarks for each molecular subgroup based on the heatmaps (percentage of infiltrated voxels and white matter infiltration) and multinomial regression analyses based on the following sample sizes: IDH-m astrocytomas (*N* 65), IDH-wt astrocytomas (*N* 54) and oligodendrogliomas (*N* 116). Age cut-off, clinical presentation, radiological images, infiltration frequency maps, distinctive BG voxels infiltrated in each subgroup and the consequent white matter infiltration maps are displayed in separate lines. The radiological images show an illustrative case for each category with axial MRI images (T2 FLAIR), with a sign of mismatch in IDH-m astrocytomas, central-posterior infiltration for IDH-wt astrocytomas and a frontal heterogeneous lesion on the right side for oligodendrogliomas. The heatmaps show infiltration frequency with hot spots in white and MNI coordinates on the *z*-axis provided below the images. Distinctive BG voxels are displayed only for statistically significant results. The specific infiltration of white matter bundles is displayed for each subgroup based on results displayed in Fig. 2 and highlighted with only the most infiltrated pathways (left IFOF and uncinate fasciculus for IDH-m astrocytomas; left IFOF and CST for IDH-wt astrocytomas; and right IFOF, FAT, SLF2-3 and arcuate fasciculus for oligodendrogliomas. The diagnosis of IDH-m astrocytomas was predicted by a younger age (<40) (AUC 0.430) compared with the other two groups (*P* < 0.05). The sensitivity and specificity for the astrocytoma IDH-m model are, respectively, 43% and 82%. IDH-wt astrocytomas displayed older age >40 years old (AUC 0.594), cognitive deficits (AUC 0.617), bilateral/central location (AUC 0.551) and the specific infiltration of the voxel A3C2S3 (AUC 0.668) as predictors (*P* < 0.05). The sensitivity and specificity for the astrocytoma IDH-wt model are, respectively, 63% and 81%. The diagnosis of oligodendrogliomas was predicted by age > than 40 years old (*P* < 0.05, AUC 0.598) and seizure onset (*P* < 0.05, AUC 0.570). The sensitivity and specificity for the oligodendroglioma model are, respectively, 59% and 76%. Multivariable prediction model outcome with PPV and NPV are provided as last row. *IDH, isocitrate dehydrogenase; m, mutation; wt, wild type; yo, years old; R, right; L, left; A, anterior; P, posterior; BG, Brain-Grid voxels*.

A summary of the multinomial regression analysis is displayed in Fig. 4 and Supplementary Table 2.

Older age (>40 years old) was two times more predictive of an oligodendroglioma diagnosis and three times more predictive of an IDH-wt diagnosis compared with IDH-m astrocytomas. Among the population older than 40 years, however, a seizure onset was three times more predictive of oligodendroglioma diagnosis compared with IDH-wt. The central or bilateral location was a predictor of IDH-wt diagnosis. The presence of cognitive impairment at onset was three times more predictive of IDH-wt diagnosis compared with the other two groups. The specific infiltration of the posterior insular sub-insular region on the left side was a distinctive predictor of IDH-wt diagnosis compared with the other two groups.

The cumulative predictive model for IDH-m astrocytomas had an AUC of 0.67 (PPV 47%, NPV 79%). The predictive model was better for IDH-wt astrocytomas, with an AUC of 0.80 (PPV 41%, NPV 82%), and oligodendrogliomas, with an AUC of 0.74 (PPV 70%, NPV 59%).

## Discussion

Our study identified multiple differences in suspected DLGG that are associated with hallmarks of DLGG subgroups. Based upon these hallmarks, we built a clinically oriented multiparametric prediction model for each subgroup. Despite multiparametric data, a prediction of the DLGG phenotype is difficult to establish from traditional clinical and radiological data available to the clinician. Still, several aspects of the suggested algorithm are worthwhile to discuss.

### Differences and hallmarks among DLGG subgroups

#### Age

Our results indicate that patients younger than 40 years old more frequently have IDH-m astrocytomas while suspected DLGG with similar radiological features in older population are more commonly IDH-wt astrocytomas or oligodendrogliomas. Some of the previous studies examining age/gender differences among glioma subtypes are based on previous WHO classifications.^12,30^ Several studies report an age-related predilection for IDH-m DLGGs, with a notable enrichment of IDH mutations observed in younger patients.^31-35^ This has been consistently documented across diverse geographic regions and patient populations, reaffirming its robustness and clinical relevance.^33-36^ The biological underpinnings driving the association between young age and IDH mutation in DLGGs remain an area of active investigation.^37,38^

The association between young age and IDH mutation in DLGGs holds significant prognostic and therapeutic implications. IDH-m DLGGs have been consistently associated with more favourable clinical outcome, including prolonged progression-free survival and OS, compared to their IDH-wt counterparts.^39-41^ Therefore, the previously suggested age cut-off for higher risk prediction in DLGG seems less robust, given the age differences among various subtypes.^37,38^

Our results are in agreement with other studies investigating the age of IDH-m oligodendrogliomas across diverse patient cohorts and geographical regions.^30,34^  ^,36,42,43^ Major reviews of the histopathological incidence on oligodendrogliomas^44,45^ demonstrated a peak incidence of IDH-m oligodendrogliomas in patients between ages 30 and 50 years^42,46,47^ but may present at even older age,^48^ with differences in the outcome that may be subtile.^43^ However, new stratification methods based on genetic body hypermethylation seems to help predict higher grade and worse outcome in older population with oligodendrogliomas.^49^

### Clinical presentation

Seizures at onset were strongly related to the oligodendroglioma diagnosis. This is in agreement with previous studies that examined the clinical features and outcomes of patients with IDH-m gliomas.^43,50-53^ In our cohort, the radiological–topographical aspects displayed a link to the seizure onset. The radiological borders displayed different risk rate in terms of seizure onset. While diffuse/infiltrative signals were linked to high risk in the IDH-wt group, bulky defined borders were associated with seizure onset in patients with oligodendrogliomas. These results may suggest, together with topographical features of the tumours subgroups, that eloquent location as well as the infiltration of specific BG voxels may facilitate seizure as a focal disconnection phenomenon.^54-56^ Interestingly, the three groups displayed different risk predictors, which have not been previously reported.

While IDH-m astrocytomas and oligodendrogliomas have been extensively studied in the literature, less attention has been directed towards understanding the clinical presentation associated with IDH-wt astrocytomas. It has been described that IDH-wt tumours display a lower rate of epileptic onset compared with IDH-m tumours.^55,57^ Our findings are concordant with others reporting more frequent cognitive impairment and neurological deficits at clinical onset in patients with IDH-wt astrocytomas.^40,52,53^ The suggested reasons for the functional difference are the impaired large-scale connectivity and loss of neuroplasticity potential due to tumour infiltration rate.^52,53^

### Tumour topography and invasive features

Several attempts have been made to suggest a sub-lobar or even a subcortical classification of DLGG using radiological overlay, deep learning models and voxel-based methods.^8,24,58,59^ We and others have shown that more systematic analysis of subcortical invasion may reflect tumour invasive features and may be able to explain neuropsychological impairment and predict outcome.^14,17,27^

A systematic preoperative analysis of patient characteristics and tumour features, including subcortical invasiveness and involvement of white matter networks, may help the neurosurgeon and oncologist individualize management and interpret the individual connectome of the patient with respect to differences in OS.^2,10,15,60^

Our results provide two different information. First, the infiltration probability for the three groups of tumours differed, in agreement with other studies using sub-lobar classification systems.^8,16-18^ IDH-m astrocytomas showed hot spots for the infiltration probability on the left side and involving the anterior insular-opercular regions. Oligodendrogliomas preferentially involve bifrontal areas with subcortical hotspots and a lateralization to the right side in this cohort. IDH-wt displayed higher infiltration of the posterior insular and temporo-parietal junctions, especially on the left side. Deep preferential location also reflected the amount and type of white matter infiltrated by each molecular subtype. For instance, the infiltration of the CST was a distinctive hallmark of the IDH-wt astrocytomas, while IDH-m astrocytomas displayed a preferential infiltration of the anterior-ventral IFOF and uncinate fasciculus. Oligodendrogliomas showed a predominant infiltration of the dorsal anterior IFOF and SLF. These results are in accordance with previous results from previous studies focusing on subcortical infiltration,^8,16-18^ reinforcing the subcortical extension as a possible hallmark among molecular subtypes of suspected DLGG at onset.

Second, we could measure tumour invasiveness based on morphological MRI sequences using the BG system. Since this system is based on cortico-subcortical landmarks, we were able to provide a quantitative measure of the subcortical extension among subgroups. The median number of BG voxels showed no clear difference among the molecular subgroups. The increasing number of BG voxels was predictive of the risk of epileptic onset in all three groups. This information is new compared with previous studies that only include the volume computation. Larger tumour volumes usually display seizures as presentation symptoms.^50,61^ We used the BG system to estimate the tumour subcortical invasiveness. In this way, we could integrate topographical information, radiological features and tumour volume, obtaining both quantitative and qualitative information. In fact, tumours with similar volume but located in deep regions such as periventricular areas showed more prominent subcortical extension with a higher number of infiltrated BG voxels.^17,24^ This sub-lobar analysis also showed that the specific invasion of A3C2S2 voxel was a predictor of epileptic onset in all three groups. This subcortical area is a crossroad of multiple large-scale networks for association pathways and projection pathways. This region is often infiltrated in diffuse gliomas^8,17,18,62^ and correlated with neurological impairment and epilepsy.^14,50,63^ A possible explanation is that the slow growth rate of the tumour may create a local cortical disconnection from the highly connected cortices, increasing the risk of epilepsy.^11,50,61^

### Prediction model of final diagnosis

The differences detected in this study among the three DLGG subgroups are summarized in Fig. 4 and Supplementary Table 2. Age, presentation symptoms and specific BG voxel position were able to predict the final diagnosis. However, the variables displayed a suboptimal predictive value (with a low sensitivity for the model fit). The cumulative prediction with multimodal variables displayed generally low positive predictive potential but higher negative prediction capacity, especially in the IDH-wt group. This is in line with other studies that displayed a maximal AUC of 0.8^64^ but worse than other models investigating two subgroups of tumours.^65^ Efforts have been made to create better model based on mixed models such as radiogenomics^66,67^ and in other studies the inclusion of connectomic-structured approach.^68^ We believe that no single model is able to precisely predict the final diagnosis of suspected DLGG, which are heterogeneous and show substantial differences in their radiological features, invasive pattern and course. The most important aspect in our study was the possibility to identify multiparametric phenotypes, integrating clinical symptoms, radiological features and sub-lobar analysis into the preoperative diagnostic algorithm. Tumour presence in specific BG voxels was one of the most sensitive single variables for predicting IDH-wt astrocytomas.

We, therefore, suggest the inclusion of a quantitative sub-lobar classification into future models for diagnosis and outcome prediction.

### The present and future of prognostic risk factors in DLGG

Patients with DLGG have historically been stratified into low and high risk as suggested by Pignatti *et al*.^69^ in 2002 and further established by the Radiation Therapy Oncology Group 9802.^70^ Age (>40 years old), tumour size (diameter > 6 cm) and neurological symptoms were included as main factors. This risk stratification is still used in a clinical context to decide when to administer chemotherapy and radiotherapy. Differences among DLGG subtypes reported in this study suggest that classical risk factors, such as being 40 years old, cannot be considered justified. The discovery of distinct molecular subtypes, such as IDH-m astrocytomas and IDH-wt astrocytomas, has significantly altered our understanding of these tumours’ biology and prognosis.^5,40,48^ In fact, IDH-m astrocytomas generally have more favourable prognosis compared to IDH-wt astrocytomas, which are now known to behave more aggressively and are genetically similar to glioblastomas.^39,41^ In our cohort age, clinical presentation and tumour topography/invasiveness differed among molecular glioma subtypes and, therefore, may be considered as confounding factors for the original risk prediction model (which included patients with unknown IDH status).

Some patients observed in clinical practice, despite older age or tumour longest diameter, have more indolent biological course due to oligodendroglioma diagnosis or complete resection of IDH-m astrocytomas when the tumour is localized in less eloquent areas. In these cases, there is growing evidence indicating that oncological treatment may be administered later during the follow-up with the same results in terms of OS^43^ or when the neuroplasticity potential is exhausted or the surgical options are not feasible anymore.^4,9^

We perceive a necessity to build new models based on prognostic risk factors in DLGG patients that consider more modern multiparametric data, such as molecular status, topography, tumour invasiveness, connectomic and cognitive symptoms.

### Strengths and limitations

The first major strength of this study is that the study population represents an unselected population-based series in a Scandinavian multicentre collaborative project. Then, our approach to study tumour topography may be considered more objective or standardized evaluation of subcortical regions. This has not been addressed in previous publications, considering that the most common approach to categorize tumours is through ‘predominant lobar infiltration’ despite the notion that subcortical extension has been proven to be a key for tumour invasion and a limiting factor for surgical resections.^27,71,72^

Our study also has some limitations, due to being a retrospective study. The variables contributing to a specific phenotype seem robust when compared to previous studies, but it should ideally be tested and further developed and improved in prospective studies.

Next, we decided to include some of the 1p19q co-deleted tumours as oligodendrogliomas, despite not being tested for IDH mutation. However, tumours with 1p19 codeletion and IDH-wt status are rare.^42,73^ Despite the fact that this aspect may create some bias in the oligodendrogliomas group regarding the hot spot infiltration and generally on topography, we believe that the effect on the final results would be minor. On the same line, we recognize that the patient data in this article come from a time interval between 2012 and 2017. The classification criteria were based on the WHO3-4 classification standard. Some of the tumours may, therefore, be classified in a different way according to WHO5. We believe that the number of mutated tumours that would have been classified as grade 4 according to WHO5 would be very discrete and not able to affect the results. In addition, patients who were re-operated and where pathology reports showed a tumour grade 4 at the first re-operation/reanalysis of the first material were excluded, as shown in Fig. 1.

The granularity of data was not always as desired. For instance, the registration of cognitive symptoms was not based on a neuropsychological assessment but on physician’s assessment of the patients. Finally, the follow-up time of this study was relatively short and therefore we decided not to analyse differences in the OS among the groups. Our aim was to better understand the course and situation of the patients at the preoperative stage, trying to predict the tumours subgroups based on multiple variables, thus presenting typical DLGG molecular phenotypes as presented in the clinic.

## Conclusions

We identified differences and specific hallmarks of molecular subgroups of suspected DLGG. The tumour subgroups based on molecular status differed in terms of age, clinical presentation and preferential location. Although predictive capabilities were modest, using multiparametric data may be relevant to build future prediction models and to better understand the onco-functional trajectory of these tumours already at the preoperative stage.

## Supplementary Material

fcaf368_Supplementary_Data

## Data Availability

The data that support the findings of this study are available on request from the corresponding author. The data are not publicly available due to privacy or ethical restrictions.
