# Supplementary material for: Unravelling differences and hallmarks in suspected diffuse low-grade gliomas: a multicentre database study
Source: Brain Commun. 2025 Sep 27;7(5):fcaf368. doi: 10.1093/braincomms/fcaf368 (PMC12508650; doi:10.1093/braincomms/fcaf368)
Supplement: fcaf368_Supplementary_Data [file fcaf368_supplementary_data.pdf]

Supplementary Material

Table 1

**BG voxel position**	Infiltration % Total Population	Infiltration Astro IDHm	Infiltration Astro IDHwt	Infiltration Oligo
BG_A1C1S1	13.8	12.3	11.1	16.4
BG_A1C1S2	25.8	23.1	18.5	31
BG_A1C1S3	17.9	18.5	16.7	18.1
BG_A1C1S4	3.8	1.5	3.7	5.2
BG_A1C2S1	19.2	21.5	13	20.7
BG_A1C2S2	35.4	33.8	35.2	37.9
BG_A1C2S3	22.9	21.5	27.8	22.4
BG_A1C2S4	4.2	4.6	0	6
BG_A1C3S1	7.1	12.3	3.7	6
BG_A1C3S2	17.5	21.5	24.1	12.9
BG_A1C3S3	7.1	6.2	13	5.2
BG_A1C3S4	1.7	1.5	0	2.6
BG_A2C1S1	20.4	18.5	14.8	24.1
BG_A2C1S2	36.7	32.3	33.3	40.5
BG_A2C1S3	20.8	23.1	22.2	19
BG_A2C1S4	3.8	3.1	5.6	3.4
BG_A2C2S1	33.8	29.2	24.1	35.3
BG_A2C2S2	51.2	49	53.7	51.7
BG_A2C2S3	27.1	26.2	37	24.1
BG_A2C2S4	5	3.1	7.4	5.2
BG_A2C3S1	11.3	16.9	9.3	9.5
BG_A2C3S2	21.3	26.2	31.5	14.7
BG_A2C3S3	7.1	6.2	11.1	6
BG_A2C3S4	1.3	1.5	0	1.7
BG_A3C1S1	17.5	20	5.6	21.6
BG_A3C1S2	35.4	38.5	24.1	37.9
BG_A3C1S3	19.2	0	29.6	13.8
BG_A3C1S4	3.8	3.1	5.6	3.4
BG_A3C2S1	32.5	40	29.6	35.3
BG_A3C2S2	55	49.2	55.6	56.9

BG_A3C2S3	25.4	18.5	50	16.4
BG_A3C2S4	5.4	4.6	13	1.7
BG_A3C3S1	12.9	15.4	13	11.2
BG_A3C3S2	22.1	23.1	31.5	16.4
BG_A3C3S3	9.2	7.7	18.5	4.3
BG_A3C3S4	0	0	0	0
BG_A4C1S1	9.2	12.3	3.7	8.6
BG_A4C1S2	25.4	30.8	22.2	22.4
BG_A4C1S3	16.7	18.5	29.6	8.6
BG_A4C1S4	2.1	1.5	5.6	.9
BG_A4C2S1	19.2	18.5	16.7	19
BG_A4C2S2	35.4	35.4	42.6	30.21
BG_A4C2S3	23.8	21.5	44.4	4.7
BG_A4C2S4	3.8	4.6	7.4	.9
BG_A4C3S1	7.5	9.2	7.4	6
BG_A4C3S2	20	21.5	27.8	14.7
BG_A4C3S3	12.9	12.3	24.1	6.9
BG_A4C3S4	0	0	0	0

The table summarizes the infiltration frequency detected for each BG voxel, for the whole cohort, and for each molecular subgroup. BG voxels with infiltration higher than 40% are highlighted in red and used for regression analyses.

Table 2

IDHm as reference category	Oligodendrogliomas			IDHwt		
**Analysed variables**	*p*	OR	CI	*p*	OR	CI
Age >40yo	.020*	2.097	1.121- 3.920	.003*	3.104	1.465- 6.576
Seizures	.063	1.800	.968 -3.349	.393	.729	.353- 1.505
Cognitive impairment	.064	.261	.063- 1.081	.020*	3.442	1.220- 9.710
Brain-Grid Voxels >8	.069	.559	.298 – 1.046	.558	1.241	.602 -2.560
Laterality L	.472	.800	.435- 1.470	.308	.686	.332- 1416
Laterality R	.433	1.277	.693- 2.352	.958	.980	.473- 2.033
Laterality B/C	.847	.836	.136- 5.138	.061	4.691	.932- 23.617
A2C1S2	.275	1.427	.754- 2.702	.906	1.048	.486-2.259
A2C2S1	.534	.820	.439- 1.533	.068	.476	.214-1.055
A2C2S2	.748	1.105	.602 -2.028	.627	1.196	.581- 2.465
A2C2S3	.763	.898	.447- 1.805	.203	1.661	.760- 3.629
A3C2S2	.321	1.361	.740- 2.504	.492	1.289	.625 – 2.660
A3C2S3	.721	1.156	.521- 2.563	<.001*	4.417	1.940- 10.057
**IDHwt as reference category**	**Oligodendrogliomas**			**IDHm**		
**Analysed variables**	*p*	OR	CI	*p*	OR	CI
Age >40yo	.245	.675	.348- 1.309	.003*	.322	.152-.682
Seizures	.007*	2.468	1.275- 4.778	.393	1.371	.664- 2.829
Cognitive impairment	<.001*	.076	.021- .278	.020*	.291	.103- .820
Brain-Grid Voxels >8	.018*	.450	.232- .873	.558	.806	.391- 1.661
Laterality L	.641	1.167	.610- 2.232	.308	1.458	.706- 3.012
Laterality R	.427	1.302	.679- 2.496	.958	1.020	.492- 2.115
Laterality B/C	.015*	.178	.044- .719	.061	.213	.042- 1.073
A2C1S2	.370	1.362	.693-2.679	.906	.955	.443- 2.058
A2C2S1	.144	1.724	.830- 3.581	.068	2.103	.947- 4.666
A2C2S2	.810	.924	.484- 1.764	.627	.836	.406- 1.722
A2C2S3	.084	.541	.269- 1.086	.203	.602	.276- 1.316
A3C2S2	.870	1.056	.551- 2.024	.492	.776	.376- 1.601
A3C2S3	<.001*	.196	.095- .405	<.001*	.226	.099 -.516
**Oligodendrogliomas as reference category**	**IDHm**			**IDHwt**		
**Analysed variables**	*p*	OR	CI	*p*	OR	CI
Age >40yo	.009*	.409	.209-.802	.642	1.188	.575-2.457
Seizures	.049*	.517	.268-.998	.006*	.359	.174-.744
Cognitive impairment	.070	3.922	.895-17.191	.001*	9.424	2.440-36.396
Brain-Grid Voxels >8	.026*	.460	.232-.909	.055	.479	.226-1.015
Laterality L	.117	.509	.191-1.355	.213	2.076	.658-6.547
Laterality R	.380	1.337	.699-2.555	.902	1.046	.509-2.152

Laterality B/C	.375	.414	.059-2.910	.126	.247	.041-1.479
A2C1S2	.208	.575	.243-1.361	.191	.539	.213-1.362
A2C2S1	.051	2.693	1.043-6.954	.895	.932	.326-2.664
A2C2S2	.740	.848	.319-2.250	.964	1.023	.379-2.763
A2C2S3	.248	1.661	.703-3.924	.432	1.446	.575-3.636
A3C2S2	.052	.470	.220-1.005	.060	.391	.147-1.041
A3C2S3	.389	1.505	.593-3.820	<.001*	9.691	3.506-26.791

The table displays the results of the multinomial regression analysis. Numerical/ continuous variables such as age and number of BG voxels were dichotomized based on ROC curves. The molecular diagnosis of the three groups was included as dependent variable while categorical/ dichotomous variables (seizure, cognitive deficits, laterality L, R and B/C, age >40 yo, number of BG voxels >8) were included as factors. The six most infiltrated BG voxels were chosen to assess a spatial prediction of the final diagnosis. A forward stepwise model was chosen and only the main effects were displayed together with the constant model.

For statistically significant results (*statistically significant for $p < 0.05$) the OR is interpreting the direction of the results. If the OR is <1 it means that the variable is linked to the probability of describing the reference group. If the OR is >1 this means that the analysed variable is predicting the analysed group and not the reference group.

In the upper part of the table the regression model was constructed with IDHm group as reference. P values, Odds ratio (OR) and Confidence Interval at 95% (CI) are displayed for oligodendrogliomas and IDHwt. All the analyzed variables were coded as categorical or dichotomous.

The middle part of the table shows the regression analysis with IDHwt group as reference. And the results are displayed for oligodendrogliomas and IDHm group. The lower part shows the regression analysis with Oligodendrogliomas as reference category and the results are displayed for IDHm and IDH wt. Older age (>40 years old) is a 2 times higher predictor of a Oligodendroglioma diagnosis and 3 times higher prediction of an IDHwt diagnosis compared with IDHm astrocytomas. Among the population older than 40 yo however, a seizure onset was 3 times higher predictive of Oligodendroglioma diagnosis compared with IDHwt. The central or bilateral location was predictor of IDHwt diagnosis. The presence of cognitive impairment at the onset was 3 times higher predictive of IDHwt diagnosis compared with the other two groups. The specific infiltration of the posterior insular sub-insular region on the left side was a distinctive predictor of IDHwt diagnosis compare with the other two groups.

It is worth noticing that the test for collinearity revealed that laterality left, right and B/C were redundant (VIF >10). Together with the sample differences among the groups this may explain the differences in the absolute values when the reference group was changed.
